# Implementation of a facilitation intervention to improve postpartum care in a low-resource suburb of Dar es Salaam, Tanzania

**DOI:** 10.1186/s13012-018-0794-x

**Published:** 2018-07-28

**Authors:** E. Pallangyo, C. Mbekenga, P. Olsson, L. Eriksson, A. Bergström

**Affiliations:** 1 School of Nursing and Midwifery, Aga Khan University, Salama House, 344 Urambo Street, P.O.BOX 38129, Dar es Salaam, Tanzania; 2 Women’s and Children’s Health, Akademiska sjukhuset, Uppsala University, SE-751 85 Uppsala, Sweden; 3 Department of Public Health and Caring Sciences, Uppsala University, Box 564, 751 22 Uppsala, Sweden; 4 Institute for Global Health, University College London, Gower Street, London, WC1E 6BT UK

**Keywords:** Intervention, Facilitation, Knowledge translation, Quality of care, Postpartum care, Tanzania, Perinatal health

## Abstract

**Background:**

Implementation of evidence into practice is inadequate in many low-income countries, contributing to the low-quality care of mothers and newborns. This study explored strategies used in a facilitation intervention to improve postpartum care (IPPC) in a low-resource suburb in Dar es Salaam, Tanzania. The intervention was conducted during 1 year in government-owned health institutions providing reproductive and child health services. The institutions were divided into six clusters based on geographic proximity, and the healthcare providers of postpartum care (PPC) (*n* = 100) in these institutions formed IPPC teams. Each team was supported by a locally recruited facilitator who was trained in PPC, group dynamics, and quality improvement. The IPPC teams reflected on their practices, identified problems and solutions for improving PPC, enacted change, and monitored the adopted actions.

**Methods:**

A qualitative design was employed using data from focus group discussions with healthcare providers (*n* = 8) and facilitators (*n* = 2), and intervention documentation. The discussions were conducted in Kiswahili, lasted for 45–90 min, were audio-recorded, transcribed verbatim, and translated into English. Thematic analysis guided the analysis.

**Results:**

Four main strategies were identified in the data: (1) *Increasing awareness and knowledge of PPC by HCPs and mothers* was an overarching strategy applied in training, meetings, and clinical practice; (2) *The mobilization of professional and material resources* was achieved through unleashing of the IPPC teams’ own potential to conduct PPC and act as change agents; (3) *Improving documentation and communication*; and (4) *Promoting an empowering and collaborative working style* were other strategies applied to improve daily care routines. The facilitators encouraged teamwork and networking among IPPC teams within and between institutions.

**Conclusion:**

This facilitation intervention is a promising approach for implementing evidence and improving quality of PPC in a low-resource setting. Context-specific actions taken by the facilitators and healthcare providers are likely integral to the successfulness of implementing evidence into practice. The results contribute to increasing the understanding of facilitation as an intervention and can be useful for researchers, HCPs, and policymakers when improving quality of postpartum care, particularly in low-income settings.

**Electronic supplementary material:**

The online version of this article (10.1186/s13012-018-0794-x) contains supplementary material, which is available to authorized users.

## Background

Globally, the mortality of mothers and newborns remains a critical challenge despite significant reductions over the past decade [1]. Sub-Saharan Africa is the most affected area, carrying 66% of the global burden of maternal deaths [1]. In Tanzania, the maternal mortality ratio is 556 deaths per 100,000 live births [2]. The main causes of maternal deaths in Tanzania, as globally, are haemorrhage, hypertensive disorders, sepsis, and unsafe abortions [3, 4]. Globally, under-five mortality has been reduced substantially [5]. However, the number of deaths due to preventable causes is still unacceptably high, especially during the neonatal period [5]. In Tanzania, the neonatal mortality rate, defined as the number of neonatal deaths per 1000 live births within the first month of life [6], is estimated at 26 deaths per 1000 live births, which contributes to 40% of all under-five deaths [2].

Globally as well as in Tanzania, postpartum care (PPC) is a neglected field in the continuum of maternal and newborn care [5]. However, the overall progress in strengthening the continuum of care, with a particular focus on the childbearing period, is promising in Tanzania. About 98% of pregnant women receive antenatal care from skilled healthcare providers (HCPs) at least once, 64% of the children are delivered by skilled birth attendants, and 75% of the children receive basic vaccinations [2]. Still, health and wellbeing assessments of mothers within 2 days after childbirth, as recommended by the WHO, remain low, with a gradual increase from 13% in 2004 [7] to 31% in 2010 [6] and 34% in 2015 [2]. The provision of PPC to newborns also remains low, with only 42% receiving PPC within 2 days of birth [2].

National policies for strengthening reproductive and child health do exist in Tanzania, with a strategic plan targeting 80% PPC coverage by 2020 [8]. The PPC national guidelines, issued in 2011, aimed to promote high-quality maternal and newborn healthcare [9]. Four PPC visits for maternal and newborn assessment are recommended: at 24 h and within 7, 28, and 42 days after childbirth [9]. Research has, however, found that HCPs lack access to these guidelines [10, 11] and there is limited supervision and feedback on practices [12]. Previous studies in Dar es Salaam [13–17] reported concerns among mothers and partners about infant feeding, sexuality, and inadequate support from HCPs. Low quality of care in health institutions is also widely reported [10, 18–21], and improvements are thus urgent. Unfortunately, strategies to increase knowledge translation, defined as the exchange, synthesis and ethically sound application of knowledge among researchers and users [22], are scarcely described. The current study describes the contextually driven strategies used in the Improving Postpartum Care (IPPC) intervention in Tanzania. Hence, this allows for the scrutiny of the intervention process, its credibility and relevance to the outcomes [23–25]. A better understanding of the strategies for knowledge translation in low-income setting is of upmost importance in facilitating evidence-based practices.

### Guiding theoretical framework for knowledge translation

There are a number of frameworks that aim to explain determinants for the successful implementation of evidence into practice [26, 27]. Most of these frameworks agree that successful implementation requires reflection on existing barriers and facilitating factors at individual and institutional levels [28]. The Promoting Action on Research Implementation in Health Services (PARIHS) framework has commonly been used to guide and understand the implementation, building on the assumption that successful implementation of evidence into practice must consider the nature of the evidence, the context in which it is to be implemented and the adopted facilitation [29]. The PARIHS posits successful implementation as a function of the nature and type of evidence (including research, clinical experience, patient experience, and local information), the qualities of the context in which the evidence is to be implemented in (including culture, leadership, and evaluation) and the way the process is facilitated [23]. According to PARIHS, the implementation of an intervention is likely to succeed when facilitators are used to empower individuals to take goal-oriented actions [23]. Facilitation is the active ingredient in this framework and involves the use of facilitators who help people to change their attitudes, habits, skills and ways of thinking and working [29]. Previous studies evaluating facilitation as an intervention in low- and middle-income countries have shown promising results in improving maternal and child health [30–33]. However, there is still a shortage of studies from various context, particular from African countries. This inspired us to focus on facilitation and use the PARIHS framework when designing and implementing the IPPC intervention in Tanzania. This study took place in a low-income country where multiple barriers exist limiting the possibilities for interventions successes. To consider the PARIHS elements and its sub-components was useful when designing the IPPC intervention.

### Setting

The IPPC intervention was quasi-experimental and used a before-and-after study design. The intervention was conducted at all government-owned health institutions providing reproductive and child health services (27 out of 29) across three levels of care in Ilala, a low-resource suburb in Dar es Salaam, Tanzania. Institutions providing reproductive and child health services (25 out of 31) across three levels of care in Temeke, a neighbouring suburb with similar characteristics in population and health care provision, were used for comparisons. Government-owned institutions are commonly accessed in Tanzania, as costs of care are more affordable compared to private institutions. Dar es Salaam is the largest commercial city in Tanzania with about 4.4 million inhabitants distributed in its three suburbs [34]. It is the most urbanized low-resource suburb in Dar es Salaam, having 1.2 million inhabitants, of which 16% live in extreme poverty [35].

In Dar es Salaam, about 50% of mothers receive PPC in the first 2 days after childbirth, which is slightly higher than the national average [2]. About 98% receive antenatal care from skilled HCPs at least once, 95% of pregnant women are delivered by skilled birth attendants, and 86% of children receive basic vaccinations [2]. Reproductive and child health services, including PPC, are provided at the different levels in government-owned health institutions: dispensaries, health centres, and suburb hospitals, regional hospitals and consultant hospitals [35].

### The IPPC intervention

Informed by the PARIHS framework [36], facilitation was adopted as the main implementation strategy to improve PPC in the 27 included health institutions between January 2015 and January 2016. It was anticipated that facilitation in Ilala would promote HCPs’ critical thinking about the barriers for improvement of PPC, reveal factors facilitating the provision of quality PPC, and develop contextually appropriate actions to enhance its provision [37]. The IPPC intervention had four phases: preparation, implementation, evaluation, and dissemination.

*The preparatory phase* encompassed various activities to enhance the researchers’ understanding of the context in which PPC is provided, to gain acceptance and make appropriate modifications as suggested by leaders and other stakeholders at different levels of the health system.

**Fig. 1:**
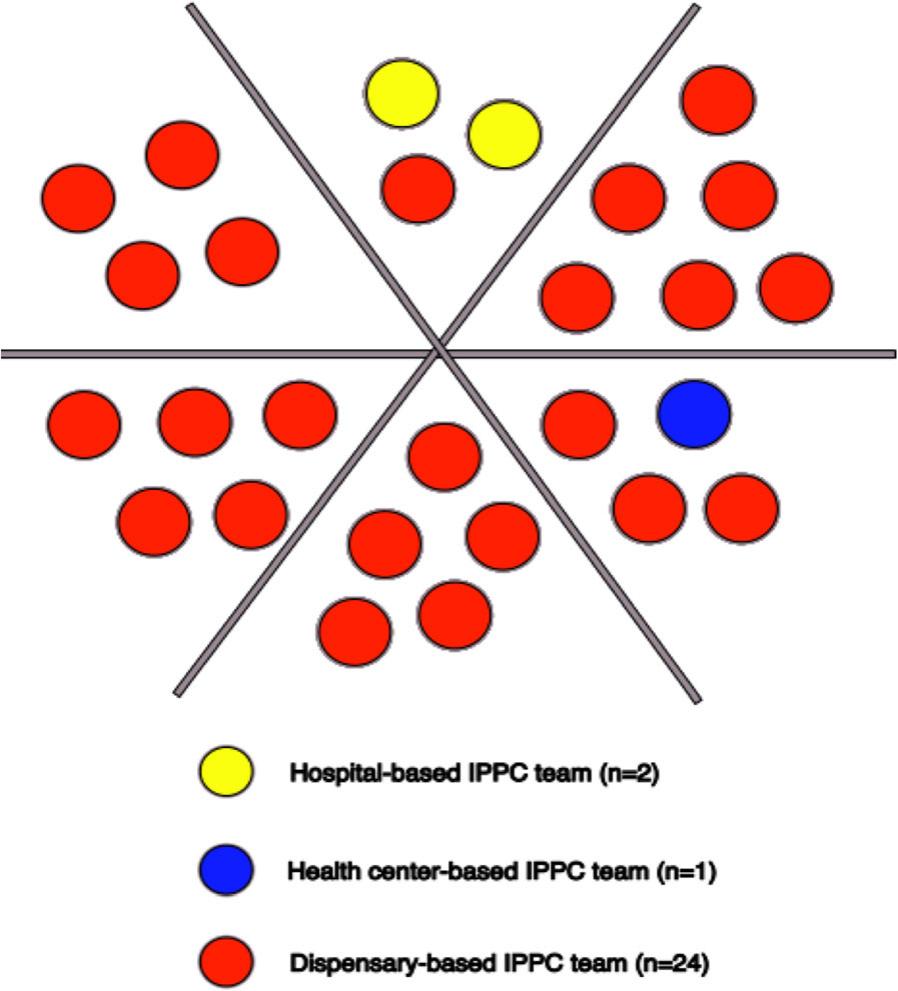
Distribution of health institutions in six clusters

Government-owned health institutions in the suburb were thereinafter grouped into six clusters based on geographic proximity. Thus, the type and number of institutions differed between the clusters (Fig. 1). One IPPC team, consisting of all HCPs involved in the provision of PPC, was formed at each of the intervention institutions. Further, one selected HCP from each of the six clusters was selected to facilitate the improvement process across the IPPC teams in that cluster. Facilitators were recruited based on the following criteria: good PPC knowledge, positive attitude to PPC, committed to improving PPC practice, and accepted and trusted by other HCPs [36]. These attributes were chosen as they are considered necessary for facilitation [36]. HCPs who met the selection criteria for facilitators were all women and registered nurse midwives: four working at different dispensaries, one at a health centre, and one at one of the hospitals. In total, the IPCC teams consisted of 100 HCPs from different professions and educational levels (Table 1).

**Table 1:** Healthcare providers by professions and institutions

Institutional level	Participants	RNM	ENM	MCHA	MO/CO
Hospitals	30	10	8	4	8
Health centre	10	2	4	2	2
Dispensaries	60	9	32	7	12
Total	100	21	44	13	22

*The implementation phase* took place over 12 months and began with a 9-day training programme with the recruited facilitators, encompassing interactive sessions in class (3 days), fieldwork (5 days), and follow-up (1 day). Topics included in the training were PPC as per the national guidelines [9], findings from research previously undertaken in the Ilala suburb [13–15, 38, 39], and how to function as a facilitator [36]. A prior developed facilitators’ guide, describing various quality improvement tools and techniques (brainstorming, nominal group technique, the Plan-Do-Study-Act (PDSA) cycle, and Strengths, Weaknesses, Opportunities and Threats analysis), was used in the training [40]. The facilitators’ guide was introduced and distributed alongside the national PPC guidelines during training. The training was led by the first author (EP), who also enacted the role of supervisor to the facilitators. The supervisor focused on empowering facilitators, who would in turn support IPPC teams to critically reflect on their practices, and identify problems and solutions for improving PPC quality. During the fieldwork, the facilitators organized meetings with representatives from the IPPC teams to introduce themselves, the IPPC intervention and PPC knowledge. The facilitators met for follow-up on the last day of training to share their experiences and to reflect upon PPC knowledge and facilitation methods.

During the implementation phase, the IPPC teams, with assistance from their facilitators, identified existing problems and solutions for PPC quality improvement at their institutions, prioritized actions for improvement, enacted change, and monitored the feasibility of the adopted actions. The improvement process was interactive and characterized by regular revision of the prioritized actions and activities as they dealt with complex and multiple barriers when improving PPC.

Representatives from the IPPC teams and their facilitator held three joint meetings in their respective IPPC cluster. In these meetings, HCPs and the facilitator shared experiences and reflected together over existing problems and solutions to improve PPC. Facilitators also visited the IPPC teams to support them in improving PPC when necessary and used phones to follow-up on their progress. IPPC teams wrote minutes from their meetings indicating identified problems, planned actions, evaluations of previous actions, and a summary of lessons learnt. The facilitators continuously kept track of IPPC teams by writing in structured diaries.

Facilitators met with the supervisor twice a month for the first 2 months and thereafter monthly. The meetings were used for monitoring and receiving the progress reports on the implementation from all the institutions. The meetings provided an opportunity for facilitators to share their experiences and to reflect on the successes, the identified problems, and solutions. The supervisor regularly documented the implementation progress and personal reflections. During the IPPC intervention, the facilitators and IPPC teams decided on what they perceived to be appropriate strategies with potential for PPC improvement. An increased understanding of such strategies is important for advancing the limited body of knowledge of facilitation interventions in low-resource settings.

*The evaluation phase* was based on data collected before and after the intervention from both the intervention institutions (*n* = 26; HCPs from one institution were absent during data collection) and the comparison institutions (*n* = 25). The population and health care characteristics of these neighbouring suburbs were similar. The data collected before the intervention revealed that no or few PPC consultations were conducted with mothers and newborns after childbirth in both the intervention and comparison institutions [41]. The data collected after the intervention showed that the intervention institutions improved HCPs’ knowledge of PPC and professional confidence, increased number of mothers at health institutions seeking for PPC and quality of care, improved PPC provision and physical resources, and that mothers’ satisfaction with PPC was high [42]. In the comparison institutions, PPC continued to be very poor [42].

### Aim of the study

The aim of this study was to explore the strategies used by facilitators and HCPs within a facilitation intervention to improve PPC in government-owned health institutions in Ilala suburb in Dar es Salaam, Tanzania.

## Methods

A qualitative design with focus group discussions (FGDs) [43] and intervention documentation [44] was used. The interactions of participants during FGDs elicited insight into the various strategies for implementation [43], while information derived from intervention documentation provided supplementary data, permitting convergence and corroboration within the FGDs [44].

### Focus group discussions and participants recruitment

A purposive sampling technique was chosen to identify participants who could provide rich answers to the research questions. Participants from varied professions who provide PPC and worked together in the IPPC team during the intervention in similar institutions were therefore selected. The FGD were conducted in institutions which had six or more HCPs who could form a large enough group to allow a fruitful interaction. FGDs were held with the IPPC teams at the mid-point and at the end-point of the intervention (Table 2).

**Table 2:** FGD participants at mid- and endpoint by profession and health institution

Midpoint FGDs	No. of participants	Endpoint FGDs	No. of participants	Professions			
				TNM	ENM	RNM	CO/MO
FGD_1_ hospital	7	FGD_6_ hospital	6	2	3	4	4
FGD_2_ hospital	7	FGD_7_ hospital	10	2	5	6	4
FGD_3_ health centre	9	FGD_8_ health centre	8	4	4	5	4
FGD_4_ dispensary	6	FGD_9_ dispensary	8	2	5	4	3
FGD_5_ facilitators^a^	6	FGD_10,_ facilitators^a^	6	0	0	6	0

Midpoint FGDs (1–4) and Endpoint FGD (6–9) were conducted with IPPC team members in June 2015 and February 2016, respectively

^a^Endpoint FGD 5 and 10 were conducted with same facilitators in February, 2016

Each group comprised 6–10 HCPs of various professions (Table 2). The FGDs with the IPPC teams were conducted at two hospitals, one health centre, and one dispensary. The FGDs with facilitators were held at the Aga Khan University at the end-point of the intervention. The mid-point FGDs focused on the outcomes and the perceptions of the intervention, and the end-point FGDs aimed to obtain in-depth information on the strategies used for implementation and the facilitators’ interaction with the IPPC teams. Out of the 31 HCPs who were invited for FGDs at the mid-point, and the 40 HCPs invited at the end, a total of four declined to participate: one due to absence from work and three due to time constraints. All six facilitators were invited and agreed to participate in the two FGDs. The participants agreed on a convenient day, time, and venue that allowed for privacy.

The FGDs were moderated by CM and a note-taker experienced in collecting field data in reproductive health. Interview guides supported the moderator during the discussions (Additional file 1). The FGDs lasted between 45 and 90 min, were conducted in Kiswahili (the national language), and were audio recorded. EP listened to the audio recordings of each FGD and discussed these with CM prior to conducting the next FGD. Thus, experience from one FGD helped to identify areas that needed further probing and assisted the moderator to strengthen the focus of the next FGDs.

### Data handling and analysis

The data comprised transcripts from 10 FGDs with IPPC teams (*n* = 8) and facilitators (*n* = 2), and intervention documentation, including minutes from meetings between the supervisor and the facilitators (*n* = 17), the supervisor’s quarterly reports (*n* = 3), and facilitators’ diaries (*n* = 6). Please note that *n* stands for the number of FGDs and documents and not the number of participants. All FGDs were transcribed verbatim, checked against recordings, and translated into English to allow non-Kiswahili-speaking researchers to contribute to the analysis and interpretation of results.

Thematic analysis, described by Braun and Clark (2012), guided the data analysis [45]. This design is suitable for the analysis of manifest and latent expressions in the data [45]. The first author (1) read the FGDs’ transcripts and the intervention documents repeatedly to familiarize herself with the data; (2) generated initial codes; (3) developed sub-themes by grouping similar codes; (4) reviewed codes and sub-themes against the entire data, carefully assessing its focus and amending possible overlaps; and (5) formed themes by relating sub-themes (Table 3). The co-authors discussed this analysis multiple times, and the process was iterative to safeguard the relevance of the themes with data. Logical and meaningful connections between sub-themes were established when writing. QRS NVivo10 computer software was used to aid movement within the data and retrieval [46].

**Table 3:** Example of thematic data analysis of transcripts from FGD with IPPC teams

Extract from FGD transcripts	Codes	Sub-theme	Theme
We [the IPPC team] could agree with colleagues and leaders locally /…/ to jointly address the challenges, example HCPs from institution (X) temporarily worked at institution (Y) to cater for the staff shortages and share PPC knowledge with colleagues	Teamwork Sharing of resources	Teamwork, networking and collaboration	Promoting an empowering and collaborative work style
…sometimes you face a challenge, which your colleagues from another institution faced earlier and solved it. For example, institution (X) could not buy a baby coat [expensive from shops] but made cheaper from local carpenter. They shared a picture and the rest who are facing similar challenge could do the same	Sharing of innovations Networking		

## Results

The analysis resulted in four themes (Table 4) as outlined below with quotes. These results illustrate that implementation encompassed several barriers and participants used different strategies that varied considerably between institutions and over the course of the intervention. Some participants were quick to engage in implementation, while others needed more time and support.

**Table 4:** Strategies used during the intervention to improve postpartum care

1. Increasing awareness and knowledge of PPC by HCPs and mothers	
2. Mobilization of professional and material resources	
3. Improving documentation and communication	
4. Promoting an empowering and collaborative work style	

### 1) Increasing awareness and knowledge of PPC by HCPs and mothers

To increase awareness and knowledge of PPC was an important strategy in training, meetings, and clinical practice. The initial facilitators’ training built confidence for raising awareness and spreading PPC knowledge in the teams and among mothers. To promote understanding and acceptance of implementing this intervention, the facilitators explained and justified the IPPC intervention to colleagues in the IPPC teams and to superiors at meetings. Increased awareness and knowledge of PPC and critical reflection allowed the facilitators and HCPs to identify their own educational needs. The supervisor was instrumental in organizing educational sessions with experts in PPC, sexuality, and mental health, which broadened HCPs’ reflections on their practices.

Now we know why breastfeeding should happen early and we educate mothers, not just asking them to breastfeed immediately after childbirth without telling them why*.* (End-point FGD with IPPC team, health centre)

Informing women and men attending health institutions for antenatal, intrapartum, and PPC about the importance and content of PPC and ideal time for visits was part of the preparation for increased attendance. In institutions where low PPC attendance persisted, the HCPs attended routine community meetings to sensitize the community members about PPC after getting permission from their superiors and the community leaders. The HCPs also encouraged mothers to share their views and suggestions for increasing the PPC attendance.

Some facilitators used educational displays produced from locally available materials to demonstrate key contents of the PPC guidelines and research results. These materials were displayed in offices and places were HCPs could easily see and read them. Similarly, HCPs displayed various educational materials in waiting areas.

I picked some information from the [PPC] guideline and place them on the noticeboard for colleagues to know about the 7^th-^, 28^th-^ and 42^nd-^ day visits. I also placed other information learnt from training about mental health, partner involvement, and HIV/AIDS*.* (End-point FGD with facilitators)

### 2) Mobilization of professional and material resources

The most prominent strategy for mobilization was the unleashing of the participants’ own potential and ability to conduct PPC and act as change agents. Further, critically reflecting on actual, ideal, and possible PPC was central to the mobilization of resources related to staffing, space, and equipment. Facilitators encouraged IPPC teams to develop innovative skills, which would enhance the utilization of existing resources and add more resources if necessary. Facilitators also encouraged colleagues to explore the possibilities to share resources within their respective health institutions and with colleagues in other institutions. Facilitators and IPPC teams actively approached superiors to mobilize staff, space, and equipment for quality PPC.

The implementation was challenged by inadequate resources, an unclear organization of physical structure, and lack of clarity about how to improve PPC among IPPC teams. Critical reflection on quality improvement revolved around organizational matters, improvising how to use the available resources, and lobbying for more resources from superiors. The creativity and ownership of the caring and the responsibility of quality improvement became vivid among HCPs who fully participated in finding new ways of dealing with challenges in their area. They were innovative and designed equipment by using cheaper alternatives and suggested modifications of available spaces at their institutions.

We tried to work on it [physical structure for PPC] yes, and put it in the budget. While waiting for a baby cot /…/, we can place a mattress on the table. When the sister [leader] comes back we can remind her*.* (Mid-point FGD with IPPC team, dispensary)

Resource mobilization for PPC was introduced on the agenda in the institutional meetings and IPPC teams strived to identify space to conduct PPC. The IPPC teams also proactively negotiated for budgeting for the missing items. These teams successfully influenced leaders to purchase equipment. Moreover, they actively approached and invited their leaders to meetings to discuss PPC matters as they considered the involvement of leaders to be key to the success of improving PPC. The meetings with leaders were used to share progress in PPC improvement, and the need for support was expressed. At institutions where leaders’ support was minimal, facilitators were invited by IPPC teams to help mobilize resources.

### 3) Improving documentation and communication

The strategies used to improve PPC practices included also improvement of the documentation of care and communication among IPPC teams within and between institutions.

Improving the documentation of care activities was described by HCPs as an important strategy to monitor the health of mothers’ and newborns. All IPPC teams agreed to keep adequate records and encouraged HCPs to support each other in case of experiencing a problem in completing the existing register books at their institutions. The need for facilitators to provide technical support on documentation for their IPPC teams was huge. Representatives from the IPPC teams also visited other institutions to learn about their documentation processes, which helped them to identify various inconsistencies in their own records. For example, there was a lack of space to record common information, such as mode of delivery, while the space for rare care activities, such as the performance of episiotomy, had more space. HCPs considered that the development of registers would have been better if they had been involved in the process and trained and supported when the records were first used.

To assist the monitoring of mothers’ migration in the suburb, the facilitators and IPPC teams jointly designed and adopted the use of referral notes. These notes were not only seen as useful for monitoring mothers but were also perceived to be a potential tool for audit. Furthermore, a contact list with representation of HCPs from the different IPPC teams was jointly developed and distributed to all the institutions to ease communication.

Two mothers having their first PPC visit to the hospital were referred to their respective dispensary. Before this, providers at the hospital communicated with HCPs at the dispensary. The two mothers were received at this dispensary and appeared on the 28^th^-day PPC visit. This dispensary was among the institutions that did not practice PPC (before the intervention)*.* (Meeting minutes, supervisor and facilitators)

Notable efforts for improving PPC were initiated from institutions where the facilitators worked. For instance, realizing that newborn health cards existed (but that they had never been used) and sensitizing IPPC teams to use them, and creating a “WhatsApp messaging application” (a messaging application for smartphones) group for easy communication. Inconsistencies and gaps in the national health information system known as “Mfumo wa Taarifa za Uendeshaji Huduma za Afya (MTUHA)” were perceived to hamper efforts for keeping records. The IPPC teams reported these problems and gave suggestions for improvement to the data coordinator in the suburb.

### 4) Promoting an empowering and collaborative work style

Collaboration within and between institutions was encouraged by facilitators and practised in various ways during the implementation of the IPPC to make the intervention participatory. The collaboration included teamwork and networking of HCPs within and across institutions in the suburb.

The collaborative strategy for working was introduced and employed during the facilitators’ training. The use of methods such as PDSA and the nominal group technique promoted participation, and the contribution of facilitators and HCPs was based on their knowledge, experiences, and reflections on the implementation of the national guidelines. The sharing of experiences and joint reflections at the supervisor and facilitators’ meetings, and between facilitators with IPPC teams, continued to be central throughout the intervention. To promote an empowering and collaborative style of working, facilitators listened attentively to the members of the IPPC teams, inviting questions and sharing experiences and providing opportunities for constructive feedback and reflection, while avoiding being judgmental.

All depended on how the flow of ideas or issues were [in the IPPC team meetings] so after knowing about the techniques to get people together [from the facilitators’ training], not being like you dominate /…/. Yeah, instead of a facilitator talking, you give the participant [HCPs] the opportunity.

Being a facilitator doesn’t mean you know everything*.* (End-point FGD with facilitators)

Role modelling turned out to be a strategy used primarily by the facilitators to inspire collaboration and constructive working relations as they all continued to work clinically as HCPs in their own workplaces throughout the intervention. Promoting change in institutions enhanced their credibility and understanding of when and how to support others. Sharing these efforts across the institutions in their geographical area functioned as a strategy, i.e., to learn from successful initiatives in other institutions, and facilitated uptake of the intervention. Three IPPC teams sent HCPs to spend some days at other institutions that had successfully developed their PPC to learn about their processes. Endorsement of constructive relations between all participants in the intervention promoted joint decision making and actions addressing contextual challenges within and across institutions.

We [facilitators] could agree with colleagues [members of IPPC teams] and leaders locally /…/ to jointly address the challenges in our institutions. For example, some HCPs from institution (X) temporarily worked at other institution (Y) to cater for the staff shortages and share knowledge with providers at those institutions*.* (Mid-point FGD with facilitators)

Decisions and actions made by IPPC teams at monthly meetings were intended to be the main pillar for the creation and up-keeping of collaboration during the intervention. However, early during the intervention, the IPPC teams and facilitators concluded that it was unrealistic to hold monthly meetings with the representation of all IPPC teams due to the shortage of staff and high workload and they, therefore, agreed to hold three meetings over the year. In addition, facilitators made monthly visits to all IPPC teams in their area and used mobile phone calls and messaging to communicate with teams for organizing meetings and follow-up and providing feedback.

Meetings were used to share the overall intervention progress, its implementation successes and barriers, feedback on performances, and possibilities to address barriers. The facilitators encouraged joint reflection and critical thinking. A facilitator could share the success and challenges of one institution and invite HCPs from another institution to learn from that success. Networking and communication promoted the sharing of experiences and innovations across IPPC teams.

Sometimes you face a challenge, which colleagues from another institution faced earlier and solved. For example, at institution (X) they could not buy a baby cot [as it was too expensive] but they made cheaper cots from a local carpenter. They later shared a picture [of baby cot on the mobile phone] and others who are facing similar challenge could do the same*.* (Mid-point FGD with facilitators)

## Discussion

This study illustrates the potential of facilitators and IPPC teams to take the lead in quality improvement in a low-resource context. They used various strategies to implement PPC national guidelines to improve the quality of PPC: increasing awareness and knowledge on PPC; mobilizing professional and material resources; improving care routines, communication, and documentation; and promoting an empowering and collaborative working style.

The implementation of the national PPC guidelines in the IPPC intervention matches the country’s priorities for improving maternal and newborn health [8], which may have stimulated its acceptance among the IPPC teams. The institutionalization of IPPC teams and the use of facilitators encouraged collaboration among HCPs and helped them develop consensus on how to handle various problems during the implementation of the intervention. The evidence in the IPPC intervention included knowledge from PPC national guidelines [9], baseline studies carried out to inform the IPPC intervention [10, 41], previous studies that had been undertaken in the study setting [13–15, 39, 47], and locally derived data. The literature shows that the nature of the evidence to be implemented determines the acceptability of the intervention as the participants are able to establish its advantages over what they are currently doing and its relevance to their work [29]. This finding is supported by previous research on the importance of clinical consensus [29] and motivation among participants [48] as enablers of successful implementation.

Advancing mothers’ awareness and PPC knowledge was a strategy used by HCPs to increase their attendance to the health services, which is fundamental in gaining their views about the service. Obtaining the perspectives of patients/clients in the PARIHS framework is considered to be a component of evidence [20]. In Tanzania, asking for mothers’ views about the care provided to them and their newborn is not common practice. Previous research in the study setting has found that mothers are unaware of what to expect [10].

Several contextual barriers are highlighted in the present study, including lack of resources, inadequate communication, lack of space, and disorganized physical structure, and were considered to affect the implementation of the IPPC intervention. Similar barriers were reported from studies in Tanzania [12, 19, 49–51] and other low-income countries in Africa [52, 53]. In southern Tanzania, irregularities in drug supplies and equipment and work overload have previously been reported to affect the provision of maternal and newborn care [19].

The existence and use of a national information system, as the present study setting indicates, is a strength for evidence-based practice. However, the current study also shows inconsistency between the practices, guidelines, and registries, which led IPPC teams to negotiate with the district data coordinator for the inclusion of missing data in MTUHA collected routinely during PPC practices. Similar inconsistencies between registries were reported in another Tanzanian study that assessed the implementation of the health information system [54]. The HCPs in both that study and the current study are concerned about the accuracy of the MTUHA data. Shortfalls in the functioning of local information systems are reported widely globally and a need to improve local data is considered a necessary step for quality improvement in health institutions [1].

The current results indicate that support from leaders was instrumental in advancing the IPPC team’s implementation efforts. However, persistently deficient support was reported from some institutions and the PPC improvement in these institutions was slow. The IPPC teams worked to mobilize help from facilitators to confront and negotiate with leadership for support. The team’s self-initiation and ability to confront and negotiate with leaders for quality improvement is likely a strength gained as a result of facilitation. This new style of working is uncommon in this context and could be difficult for HCPs to undertake. Nevertheless, the team’s hard work was appreciated by some leaders, who thereafter supported them. This can be described as providers gaining insights and demonstrating increased knowledge and professional confidence [42], thus taking control of their own practices. They could be also challenging, and this may portray the dominant top-down authoritative style of leadership in the health system which promotes HCPs’ culture of waiting to be told what to do [10, 55]. This culture limits innovations for identifying problems and solutions to barriers in their daily practices. Studies from other low-income countries also show that leadership support is influential in implementing evidence into practice [52, 53, 56]. Similar to the present study, a study in Uganda on knowledge translation indicated that the leadership support of HCPs was inadequate, sometimes of a dominant nature, and this was a common obstacle to knowledge translation in this setting [52]. Contrasting the leadership style in the present study, transformational leaders promote openness and reflections and support subordinates’ efforts towards achieving their goals [29]. A shift towards this leadership style could release the potential of the HCPs in addressing implementation barriers and improving quality of care in health institutions in this study setting.

The findings of this study indicate that collaboration and teamwork among IPPC teams were strengthened in the course of the intervention, particularly as teams increasingly engaged in problem-solving activities and other efforts supported by the facilitators. Similar results are described in a systematic review of teamwork effectiveness, which indicates that teamwork in various contexts, including healthcare, improves performance, particularly alongside adequate preparation (e.g. setting goals and actions), support (by facilitators, in our case, and leaders), and reflection on performance and feedback [57]. The review also indicates that teamwork is strengthened through practices and not merely through any educational lectures that may be provided [57]. Another systematic review indicates that teamwork contributes to the quality of care improvement through collaboration and cohesiveness among HCPs [58]. However, HCPs’ differences in perceptions about teamwork contribute to teams’ ineffectiveness [59]. Obtaining a better understanding of the teams’ characteristics, clarification of differences, and facilitation of its improvement are suggested [58]. Our results indicate that facilitation is of paramount importance in this context for enabling a team’s efforts.

### Limitation of the study

The 1-year duration for the implementation of this intervention was short, given the contextual realities of multiple barriers and introducing the HCPs to a new approach to working. Devoting more time would have given room for the conceptualization of the implementation. Although PDSA was appreciated and applied as a quality improvement method, the use of this tool was challenging for IPPC teams and the data show that the work of the IPPC teams did not follow its systematic structure. This may suggest that providing more support and time is required to acquaint HCPs with PDSA. [27]. Although the design adopted in the IPPC intervention has limitations, the current study is not comparative and only aims to gain an understanding of the functioning of the facilitation intervention in the study setting.

## Conclusion

The facilitation intervention adopted in this project is a promising approach for implementing evidence into practice and improving quality of PPC in a low-resource setting. The context-specific nature of the actions taken by the facilitators and HCPs are likely to be key for evidence to be successfully implemented into practice. The use of facilitators who have knowledge and experience of the context seems to have potential in simplifying the identification of barriers and strategies for implementation. The results of this study may be transferable to other contexts to inform the implementation of evidence in reproductive healthcare practices in low- and middle-income countries. This study contributes to the scientific body of knowledge on the experiences of implementation of evidence-based interventions in low-income setting.

## Additional file

Additional file 1: Focus group discussions guides (DOCX 14 kb)
