# Supplementary material for: Implementation of a facilitation intervention to improve postpartum care in a low-resource suburb of Dar es Salaam, Tanzania
Source: Implement Sci. 2018 Jul 28;13:102. doi: 10.1186/s13012-018-0794-x (PMC6064049; doi:10.1186/s13012-018-0794-x)
Supplement: Supplementary file 1 — Focus group discussions guides (DOCX 14 kb) [file 13012_2018_794_MOESM1_ESM.docx]

**Additional file 1: Focus group discussions guides**

1. *1^st^ FGD guide with facilitators*

- **What is your experience of the intervention to improve PPC?** What was done and what facilitators perceived about the outcome of the intervention
- **What do you think about the sustainability of this intervention model?** What would make it sustainable?
- **Would it be possible to use this intervention model in other areas?** Why/why not?
- **What would be needed to adjust in this model of care for its refinement?**

1. *2^nd^ FGD guide with facilitators (Follow up)*

- **How would you describe the training conducted at the beginning of this project?** What are your perceptions on how the training developed your capacity as a facilitator?
- **How would you describe your role in the project?**(To increase awareness to colleagues about change of PPC, assisting groups to set goals, organizing and facilitating meetings, gathering and disseminating information, disseminating knowledge to providers and mothers, building relationships and communication?)
- **Could you describe how a typical meeting with supervisor (EP) was performed?** (How was it structured? What did you discuss? How did the supervisor support the implementation of the project? Did you mainly talk about PPC or did you also talk about how you could enact your role as a facilitator?)
- **Let’s move on. Can you now elaborate around the meetings with IPPC teams from the health institutions in your centre?** (How did you act during these meetings and what actually happened? Did you use any particular methods (e.g. brainstorming, PDSA)? How did you perceive your role in the group in comparison to the participants? What was easy and what were the challenges? How did you solve problems during the facilitation process?)
- **Quite early in the project you started to visit individual health institutions, could you describe such a meeting?** (Was that similar to the other meetings or what actually happened here, e.g. did you use brainstorming or PDSA or did you mainly give advice on PPC? Did you mainly talk to individuals or discuss with a group of people?
- **How would you describe a good facilitator based on your experiences?** (What would you advice a coming facilitation intervention project leaders to keep, improve or skip, based on your experiences?

1. *FGD guide with healthcare providers*

- **What is your experience of the intervention to improve PPC?** What was done and what healthcare providers perceived about the intervention
- **What do you think about the sustainability of this intervention model?** What would make it sustainable?
- **Would it be possible to use this intervention model in other areas?** Why/why not?
- **What would be needed to adjust in this model of care for its refinement?**
